# Effect of *Helicobacter Pylori* Eradication on Human Gastric Microbiota: A Systematic Review and Meta-Analysis

**DOI:** 10.3389/fcimb.2022.899248

**Published:** 2022-05-04

**Authors:** Yang Guo, Xue-Shan Cao, Guan-Yi Guo, Meng-Ge Zhou, Bo Yu

**Affiliations:** ^1^ Department of Dermatology, Institute of Dermatology, Peking University Shenzhen Hospital, Shenzhen Peking University-The Hong Kong University of Science and Technology Medical Center, Shenzhen, China; ^2^ College of Life Science and Oceanography, Shenzhen University, Shenzhen, China; ^3^ Department of Hematology, the Second Hospital of Hebei Medical University, Key Laboratory of Hematology, Shijiazhuang, China; ^4^ Vanke School of Public Health, Tsinghua University, Beijing, China

**Keywords:** *Helicobacter pylori*, gastric microbiota, eradication, meta-analysis, humans

## Abstract

**Background:**

*Helicobacter pylori* (*H. pylori*) infection is a major risk factor for gastric cancer and eradication of *H. pylori* is recommended as an effective gastric cancer prevention strategy. The infected individuals show microbial dysbiosis of gastric microbiota. In recent years, agrowing number of studies have focused on gastric microbiota changes following *H. pylori* eradication. In the present study, we aim to evaluate the influence of successful *H. pylori* eradication on the short-term and long-term alterations of human gastric microbiota using a method of systematic review and meta-analysis.

**Methods:**

We did a systematic search based on three databases (PubMed, EMBASE, and Web of Science) in November 2021. Additional articles were also identified by reviewing references cited in the included papers. Human studies that reported changes in gastric microbiota following successful *H. pylori* eradication were enrolled. PROSPERO registration number: CRD42021293796.

**Results:**

In total, nine studies enrolling 546 participants were included. Regarding quadruple therapy, alpha diversity indexes increased within 1 month after eradication; significant differences in gastric microbial community structure between before and after eradication were also seen within 1 month. The trends of the above-mentioned diversity changes persisted with a follow-up of 6 months. The microbial composition altered significantly after eradication and the relative abundance of *H. pylori*-related taxa decreased. Accordingly, gastric commonly dominant commensals were enriched. Bioinformatic analyses of microbiota functions showed that bacteria reproduction-related pathways were down-regulated and pathways of gastric acid secretion, etc. were up-regulated. For triple therapy, similar trends of alpha diversity and beta diversity changes were observed in the short-term and long-term follow-up. Also, after eradication, *H. pylori* was not the gastric dominant bacteria and similar changes in gastric microbial composition were found. For gastric microbial interactions, a decrease in microbial interactions was seen after eradication. Additionally, regarding whether successful *H. pylori* eradication could restore gastric microbiota to uninfected status, the results remain controversial.

**Conclusion:**

In conclusion, successful *H. pylori* eradication could reverse the gastric microbiota dysbiosis and show beneficial effects on gastric microbiota. Our findings may provide new insight for exploring the role of *H. pylori* and the whole gastric microbiota in gastric carcinogenesis.

## Introduction

*Helicobacter pylori* (*H. pylori*) infection is an important risk factor for gastric cancer (GC), which is the third leading cause of cancer-related death in the world (Polk and Peek, 2010; Bray et al., 2018). *H. pylori* was classified by the World Health Organization International Agency for Research on Cancer (WHO-IARC) as a type I carcinogen (WHO-IARC, 1994). The *H. pylori* eradication therapy is effective in reducing the risk of GC and precancerous lesions (You et al., 2006; Ma et al., 2012; Ellis et al., 2019). Accordingly, *H. pylori* eradication has been recommended by WHO-IARC as an effective prevention strategy for GC (WHO-IARC, 2014).

Evidence has shown that *H. pylori* plays a critical role in the development of GC. *H. pylori* infection could lead to chronic inflammation of gastric mucosa and subsequent histopathological changes in the gastric epithelium, promoting the occurrence of precancerous gastric lesions (Díaz et al., 2018). However, other organisms may also contribute to gastric carcinogenesis. According to the animal studies (Lofgren et al., 2011) using hypergastrinemic insulin-gastrin (INS-GAS) transgenic mice, gastric lesions take longer to develop in germfree INS-GAS mice than the SPF INS-GAS mice; compared with *H. pylori*-infected INS-GAS mice with complex gastric microbiota, *H. pylori* monoassociation caused less severe gastric lesions and delayed onset of gastrointestinal intraepithelial neoplasia. These findings suggest that non-*H. pylori* microbes and/or their interactions might promote gastric lesions and even GC. Thus, it is important to assess the role of the whole gastric microbiota in gastric carcinogenesis (Scott et al., 2019).

For *H. pylori*-infected gastric microbial community, *H. pylori* dominate the microbiota in the stomach and the gastric microbiota dysbiosis is formed accordingly. The major characteristics of *H. pylori* infection-induced gastric microbiota dysbiosis include reduced microbial diversity, altered microbial community structure, composition, bacterial interactions, etc. (Maldonado-Contreras et al., 2011; Coker et al., 2017; Parsons et al., 2017; Schulz et al., 2018). The influence of *H. pylori* eradication on gastric microbiota has received increased attention and there are increasing studies focused on this issue and whether post-eradication gastric microbiota restored to uninfected status; controversial findings were reported. The recommended strategies for *H. pylori* eradication include traditional bismuth quadruple therapy, concomitant non-bismuth quadruple therapy, proton pump inhibitor (PPI) triple therapy, etc. (Fallone et al., 2016; Malfertheiner et al., 2017); evaluation of the influence of different strategies for *H. pylori* eradication on gastric microbiota is limited. Therefore, it is warranted to summarize the available literature for understanding the effect of *H. pylori* eradication on gastric microbiota.

In the present study, we investigated the influence of successful *H. pylori* eradication on human gastric microbiota in the short-term and long-term using a method of systematic review and meta-analysis. Our findings may provide new insight for exploring the role of *H. pylori* and the whole gastric microbiota in gastric carcinogenesis.

## Methods

The present study was designed and reported according to the recommendations of the Preferred Reporting Items for Systematic Reviews and Meta-Analyses (PRISMA statement) (Moher et al., 2009). The study was registered with the PROSPERO database (Registration number: CRD42021293796).

### Literature Search

We did a systematic search using three electronic databases, including PubMed, Web of Science, and EMBASE, on November 27, 2021. The search strategy is a combination of parameters “microbiome”, “microbiota”, “microflora”, “bacterial flora”, “bacterial community”, “*Helicobacter pylori*”, “*H. pylori*”, “eradication”, “treatment”, and “therapy”. The full search strategy is in **Supplementary Table S1**. Additionally, other data sources were also considered: such as reviewing references cited in the included papers.

### Study Selection and Data Extraction

The eligibility criteria for study inclusion were established according to the PICOS strategy:

Participants/population: individuals with *H. pylori* infection.

Interventions: successful *H. pylori* eradication.

Comparators/controls: gastric microbiota before successful *H. pylori* eradication *v.s.* gastric microbiota after successful *H. pylori* eradication.

Outcomes: primary outcome: changes of gastric microbiota diversity (alpha diversity and beta diversity) and treatment-related differential microbes; secondary outcome: alterations of gastric microbial interactions and microbiota functions.

Study design: interventional studies with gastric microbiota evaluated using 16S rRNA gene sequencing.

Furthermore, no language restrictions were applied. Conference abstracts were excluded as limited information was reported. Two researchers (GY and XSC) independently did the selection process and the data extraction; the data analyses were performed by GY and GYG independently. Discrepancies were resolved through the group discussions.

For the included articles, using pre-defined data extraction form (**Supplementary Table S2**), two researchers independently extracted information, with any disagreements resolved by group discussions. The following information were extracted: I. Basic information of included studies (authors, publication year, journal, title, etc.), II. Interventions and comparisons (*H. pylori* eradication therapy and follow-up time), III. Outcomes (major findings about alpha diversity, major findings about beta diversity, major findings about differential microbes, etc.), and IV. Information of data extraction (reviewer name and date of data extraction).

### Study Quality Assessment

Methodological Index for Non-Randomized Studies (Slim et al., 2003) (MINORS, **Supplementary Table S3**) was used to assess the quality of included non-randomized studies. The MINORS consists of 12 indexes: 1) a clearly stated aim, 2) inclusion of consecutive patients, 3) prospective collection of data, 4) endpoints appropriate to the aim of the study, 5) unbiased assessment of the study endpoint(s), 6) a follow-up period appropriate to the aim of the study, 7) loss to follow-up less than 5%, 8) prospective calculation of the study size, 9) an adequate control group, 10) contemporary groups (control and studied group should be managed during the same time period, no historical comparison), 11) baseline equivalence of groups and 12) an adequate statistical analyses. The items were scored 0 if not reported; 1 when reported but inadequate; and 2 when reported and adequate. Studies were considered as high quality if the total score was ≥17, medium quality if the total score was 9∼16, and low quality if the total score was <9. In addition, for randomized studies (if any), the Cochrane risk-of-bias tool for randomized trials (RoB 2) (Minozzi et al., 2020) (**Supplementary Table S4**) will be used. The RoB 2 tool is structured into five domains, including 1) bias arising from the randomization process, 2) bias due to deviations from intended interventions, 3) bias due to missing outcome data, 4) bias in measurement of the outcome, and 5) bias in the selection of reported result; a series of signaling questions were asked in the five domains. Based on the answers to the signaling questions, an overall evaluation of bias will be given including “low risk of bias”, “some concerns” or “high risk of bias”.

### Statistical Analysis

Data syntheses were focused on alpha diversity indexes. In detail, only studies using the same index of alpha diversity were included for meta-analysis. The variables were expressed as mean ± standard deviation (SD) for further calculation; for variables expressed as median and interquartile range (IQR), they were converted into mean with SD through a recommended formula (Wan et al., 2014); the weighted mean differences (WMDs) with 95% confidence intervals (CIs) were calculated for alpha diversity indexes (Ye et al., 2020). In addition, the heterogeneity across the studies was assessed by determining the I^2^ statistic to quantitatively measure the inconsistency across studies.; the heterogeneity across studies was identified if the I^2^ >50%. The fixed-model (if heterogeneity was not detected) or random model (if heterogeneity was detected) will be used.

Subgroup analyses were conducted according to types of therapy, country, agents, and follow-up period. The data analyses in our study were performed using Review Manager (version 5.4).

## Results

**Figure 1:**
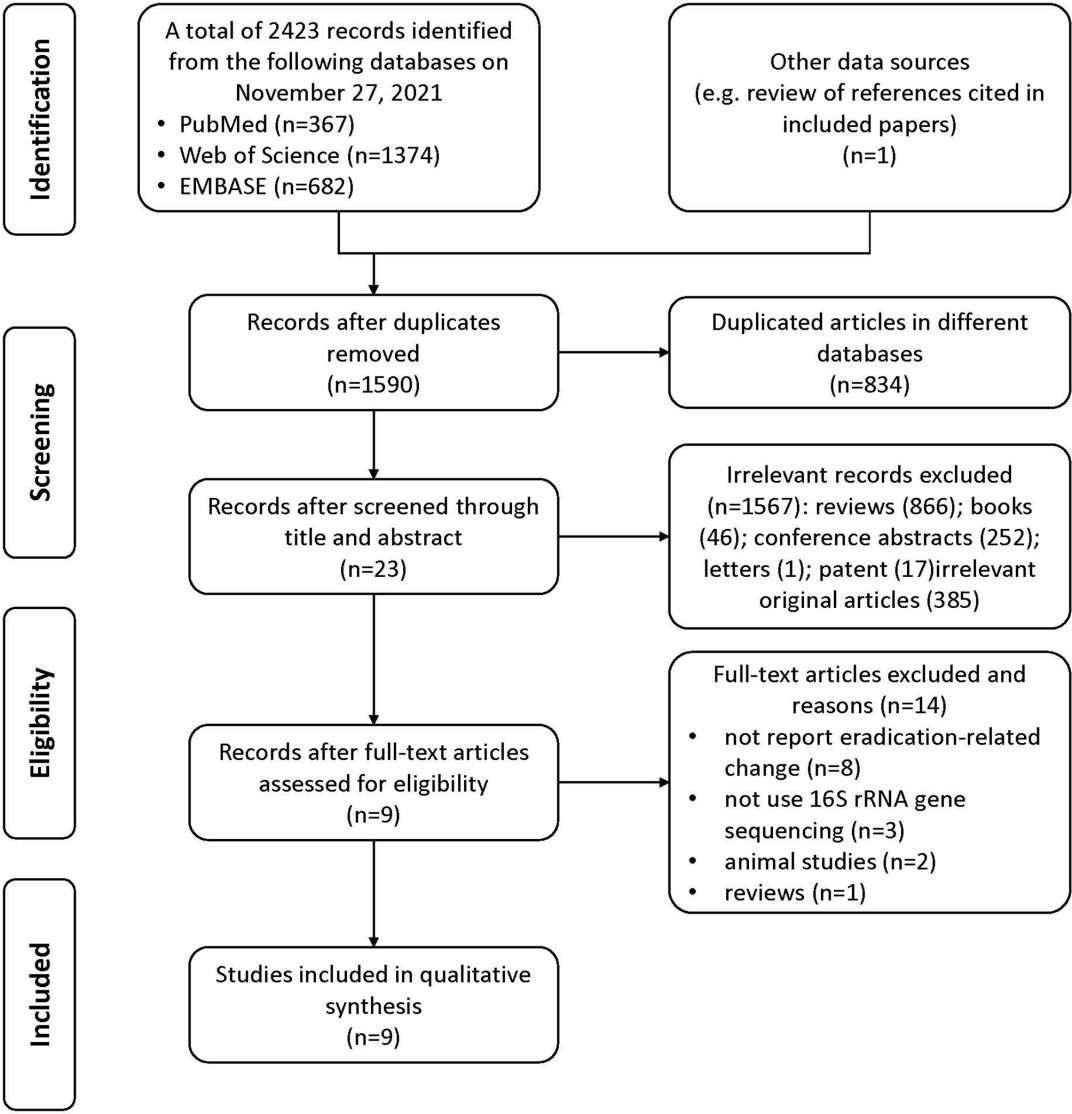
Flow chart of literature identification and selection process.

In total, 2423 records were identified from the three databases (PubMed, Web of Science, and EMBASE) and one record was identified by review of references cited in included papers; 834 of them were repeatedly included from more than one search database and were excluded. Finally, nine articles (Li et al., 2017; He et al., 2019; Serrano et al., 2019; Guo et al., 2020; Shin et al., 2020; Sung et al., 2020; Mao et al., 2021; Watanabe et al., 2021; Yuan et al., 2021) with 546 participants were included in the present study. The study selection process is shown in the PRISMA flow diagram (**Figure 1**).

### Characteristics of Included Studies

The detailed characteristics of the included nine studies are shown in **Table 1**. Six (Li et al., 2017; He et al., 2019; Guo et al., 2020; Sung et al., 2020; Mao et al., 2021; Yuan et al., 2021) of them were conducted in China. For the participants, eight (8/9) studies focused on adults (Li et al., 2017; He et al., 2019; Guo et al., 2020; Shin et al., 2020; Sung et al., 2020; Mao et al., 2021; Watanabe et al., 2021; Yuan et al., 2021). Regarding the therapy of *H. pylori* eradication, there were four (He et al., 2019; Guo et al., 2020; Mao et al., 2021; Yuan et al., 2021) studies that evaluated quadruple therapy and five studies (Li et al., 2017; Serrano et al., 2019; Shin et al., 2020; Sung et al., 2020; Watanabe et al., 2021) that focused on triple therapy. For the follow-up duration, these studies reported short-term (< 6 months) (Li et al., 2017; He et al., 2019; Serrano et al., 2019; Mao et al., 2021; Yuan et al., 2021) and long-term (≥ 6 months) (He et al., 2019; Guo et al., 2020; Shin et al., 2020; Sung et al., 2020; Watanabe et al., 2021) influence of *H. pylori* eradication.

**Table 1:** Characteristics of the included studies.

No.	Study	Region	Participants	Therapy	Follow-up	Total sample size
1	(Yuan et al., 2021)	China	Adults	Quadruple therapy (amoxicillin 1000 mg + clarithromycin 500 mg + esomeprazole 20 mg + potassium bismuth citrate 200 mg, twice daily for 14 days) Probiotics supplemented quadruple therapy (amoxicillin 1000 mg + clarithromycin 500 mg + esomeprazole 20 mg + potassium bismuth citrate 200 mg, twice daily for 14 days) Probiotics monotherapy	2 months	151
2	(Watanabe et al., 2021)	Japan	Adults	Triple therapy (a PPI [esomeprazole, lansoprazole, or rabeprazole] or vonoprazan + amoxicillin + clarithromycin, twice daily for 7 days)	13 months	29
3	(Mao et al., 2021)	China	Adults	Quadruple therapy (omeprazole 20 mg + bismuth pectin 200 mg + furazolidone 100 mg + amoxicillin 1000 mg, twice daily for 14 days)	4 weeks	63
4	(Sung et al., 2020)	China	Adults	Triple therapy (omeprazole 20 mg + amoxicillin 1g + clarithromycin 500mg, twice daily for 7 days)	1 year	102
5	(Shin et al., 2020)	Korea	Adults	Triple therapy (a standard dose of PPI + amoxicillin 1g + clarithromycin 500 mg, twice daily for 7-14 days)	57.4 months	32
6	(Guo et al., 2020)	China	Adults	Quadruple therapy (omeprazole 20 mg [twice daily for 10 days] + tetracycline 750 mg [three times daily for 10 days] + metronidazole 400 mg [three times daily for 10 days] and bismuth citrate 300 mg [twice daily for 10 days])	6 months	107
7	(Serrano et al., 2019)	Chile	Children	Triple therapy (amoxicillin + clarithromycin + omeprazole, for 14 days)	2 months	12
8	(He et al., 2019)	China	Adults	Quadruple therapy (esomeprazole 20 mg + bismuth subcitrate 220 mg + amoxicillin 1g + furazolidone 100 mg, twice daily for 14 days)	6 weeks 26 weeks	17
9	(Li et al., 2017)	China	Adults	Triple therapy (esomeprazole 20 mg + amoxicillin 1g + clarithromycin 500 mg, twice daily for 7 days)	8 weeks	33

### Quality Assessment of Included Studies

**Figure 2:**
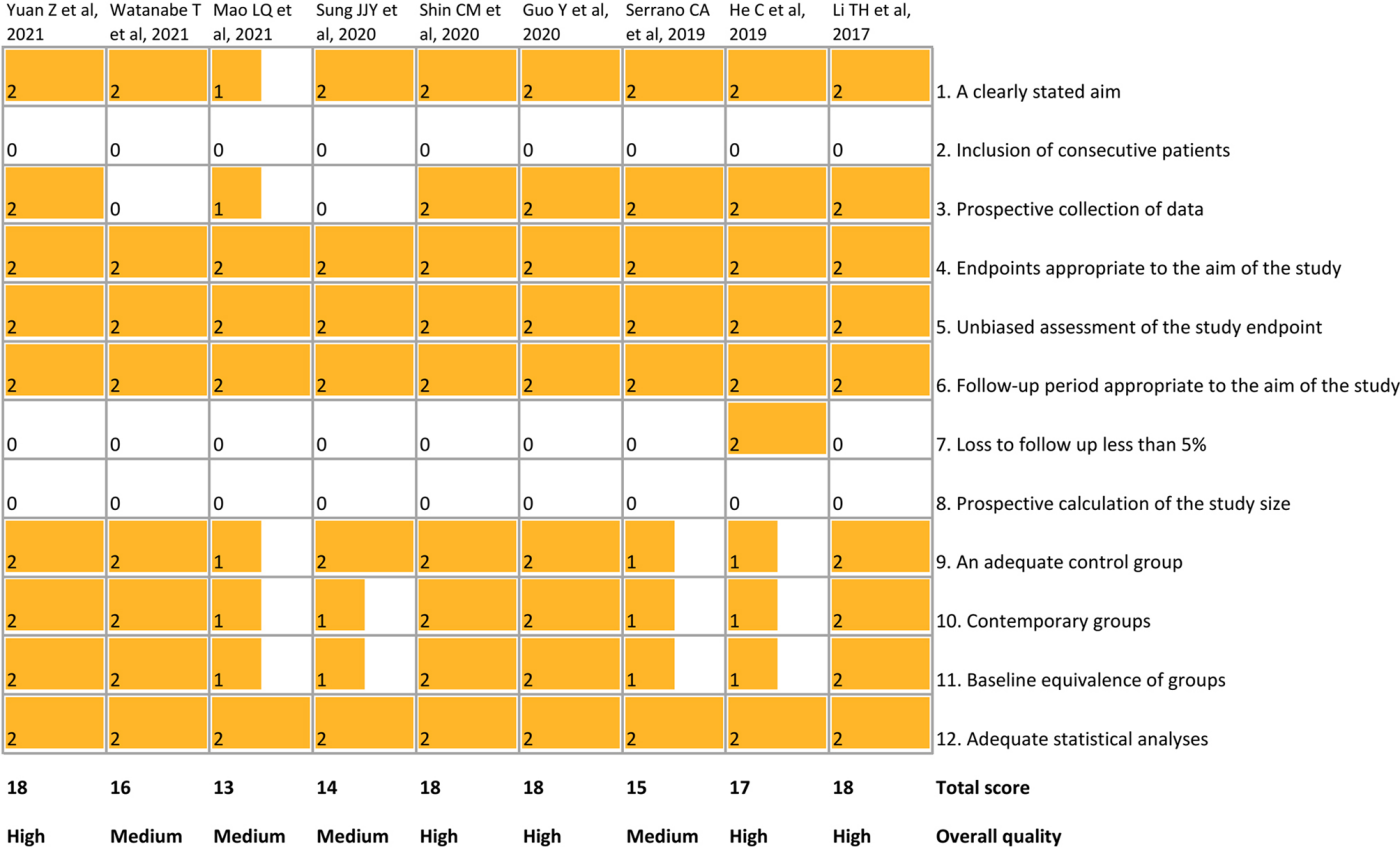
Quality assessment of included studies using MINORS. MINORS, Methodological Index for Non-Randomized Studies.

The methodological quality of the included studies is shown in **Figure 2**. The total scores ranged from 13 to 18. Five studies (Li et al., 2017; He et al., 2019; Guo et al., 2020; Shin et al., 2020; Yuan et al., 2021) were considered to be high-quality studies.

### Effect of *H. pylori* Eradication on Gastric Microbiota Diversity

The individual studies and summary results are shown in **Table 2**. According to the studies focusing on quadruple therapy which reported findings on alpha diversity, alpha diversity indexes increased within 1 month after eradication, including Shannon, Simpson, observed species, and Chao 1 (Mao et al., 2021); significant differences in gastric microbial community structure (beta diversity) between before and after eradication were also seen within 1 month (Mao et al., 2021). For longer follow-up, the trends of above-mentioned diversity changes persisted with follow-up of 6 months: higher alpha diversity indexes were seen compared to baseline, including Shannon (He et al., 2019; Guo et al., 2020), richness index (Guo et al., 2020), Chao 1 (He et al., 2019), and Sobs index (He et al., 2019); significant differences of beta diversity were also observed compared with that at baseline (He et al., 2019; Guo et al., 2020).

**Table 2:** Major findings of the included studies on alpha and beta diversity of gastric microbiota.

No.	Study	Therapy	Follow-up	Changes of alpha diversity of gastric microbiota	Changes of beta diversity of gastric microbiota
1	(Yuan et al, 2021)	Quadruple therapy (amoxicillin 1000 mg + clarithromycin 500 mg + esomeprazole 20 mg + potassium bismuth citrate 200 mg, twice daily for 14 days)	2 months	Shannon: ↑, Simpson: ↑	Significant differences were reported after eradication
		Probiotics supplemented quadruple therapy (amoxicillin 1000 mg + clarithromycin 500 mg + esomeprazole 20 mg + potassium bismuth citrate 200 mg, twice daily for 14 days)	2 months	Shannon: ↑, Simpson: ↑	Significant differences were reported after eradication
		Probiotics monotherapy	2 months	Shannon: NS, Simpson: NS	Significant differences were reported after eradication
2	(Watanabe et al, 2021)	Triple therapy (a PPI [esomeprazole, lansoprazole, or rabeprazole] or vonoprazan + amoxicillin + clarithromycin, twice daily for 7 days)	13 months	Shannon: ↑, observed OTUs: NS, ACE: NS, Chao1: NS	Significant differences were reported after eradication
3	(Mao et al, 2021)	Quadruple therapy (omeprazole 20 mg + bismuth pectin 200 mg + furazolidone 100 mg + amoxicillin 1000 mg, twice daily for 14 days)	4 weeks	observed species: ↑, Chao1: ↑, Shannon: ↑, Simpson: ↑	Significant differences were reported after eradication
4	(Sung et al, 2020)	Triple therapy (omeprazole 20 mg + amoxicillin 1g + clarithromycin 500mg, twice daily for 7 days)	1 year	Shannon: ↑	Significant differences were reported after eradication
5	(Shin et al, 2020)	Triple therapy (a standard dose of PPI + amoxicillin 1g + clarithromycin 500 mg, twice daily for 7-14 days)	57.4 months	Shannon: ↑, phylogenetic diversity: ↑	Significant differences were reported after eradication
6	(Guo et al, 2020)	Quadruple therapy (omeprazole 20 mg [twice daily for 10 days] + tetracycline 750 mg [three times daily for 10 days] + metronidazole 400 mg [three times daily for 10 days] and bismuth citrate 300 mg [twice daily for 10 days])	6 months	Shannon: ↑, richness index: ↑	Significant differences were reported after eradication
7	(Serrano et al, 2019)	Triple therapy (amoxicillin + clarithromycin + omeprazole, for 14 days)	2 months	Shannon: ↑, Simpson: ↑	Not reported
8	(He et al, 2019)	Quadruple therapy (esomeprazole 20 mg + bismuth subcitrate 220 mg + amoxicillin 1g + furazolidone 100 mg, twice daily for 14 days)	6 weeks	Shannon: ↑, Sobs index: NS, Chao 1: NS	Significant differences were reported after eradication
			26 weeks	Shannon: ↑, Sobs index: ↑, Chao 1: ↑	Significant differences were reported after eradication
9	(Li et al, 2017)	Triple therapy (esomeprazole 20 mg + amoxicillin 1g + clarithromycin 500 mg, twice daily for 7 days)	8 weeks	Shannon: ↑, phylogenetic diversity: ↑	Not reported

NS, not significant; ↑, increased.

In terms of triple therapy, similar trends of alpha diversity and beta diversity change were observed in the long-term (≥ 6 months) follow-up (**Table 2**). The triple therapy led to an increase of alpha diversity indexes in the short-term, including Shannon (Li et al., 2017; Serrano et al., 2019) and phylogenetic diversity (Li et al., 2017), and in the long-term, including Shannon (Sung et al., 2020; Watanabe et al., 2021) and phylogenetic diversity (Shin et al., 2020). Similar alterations of differences in beta diversity were reported (Shin et al., 2020; Sung et al., 2020; Watanabe et al., 2021), as well.

**Figure 3:**
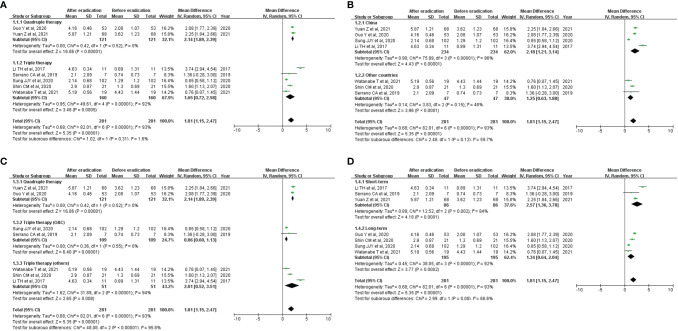
Effect of successful *H. pylori* eradication on Shannon index. **(A)** Subgroup analysis according to types of therapy; **(B)** Subgroup analysis according to countries; **(C)** Subgroup analysis according to agents; **(D)** Subgroup analysis according to follow-up period. Forest plots describing weighted mean difference (WMD) with 95% confidence interval (CI) for included studies reporting Shannon index. OAC, omeprazole + amoxicillin + clarithromycin.

The meta-analysis results are shown in **Figure 3**. Overall, the Shannon index increased significantly after eradication of *H. pylori* (WMD=1.81 [1.15, 2.47]); marked high heterogeneity was seen (overall I^2 =^ 93%). In the subgroup analysis, results of subgroup analyses according to types of therapy (quadruple therapy *vs.* triple therapy), country (China *vs.* others), and follow-up period (short-term *vs.* long-term) were consistent with overall result (**Figures 3A, B, D**). In addition, omeprazole-based triple therapy was inconsistent with the overall results (**Figure 3C**).

### Effect of *H. pylori* Eradication on Gastric Microbial Composition

The altered taxa at phylum level and at genus level are presented in **Table 3**. The overall findings according to the enrolled studies are summarized as follows. Overall, the gastric microbial composition altered significantly after quadruple therapy or triple therapy. Specifically, the relative abundance of *H. pylori*-related taxa decreased at different levels, such as *Proteobacteria* (phylum) and *Helicobacter* (genus) (Li et al., 2017; He et al., 2019; Guo et al., 2020; Shin et al., 2020; Sung et al., 2020; Mao et al., 2021; Watanabe et al., 2021; Yuan et al., 2021) (**Table 3**). Accordingly, gastric commonly dominant commensals were enriched following eradication, such as *Firmicutes*, *Bacteroides*, *Actinobacteria*, etc. (He et al., 2019; Guo et al., 2020; Shin et al., 2020; Mao et al., 2021; Watanabe et al., 2021; Yuan et al., 2021) (**Table 3**).

**Table 3:** Major findings of gastric microbial composition.

No.	Study	Therapy	Follow-up	Change of gastric microbial composition
1	(Yuan et al, 2021)	Quadruple therapy (amoxicillin 1000 mg + clarithromycin 500 mg + esomeprazole 20 mg + potassium bismuth citrate 200 mg, twice daily for 14 days)	2 months	↓: *Proteobacteria; Helicobacter* ↑: *Firmicutes, Bacteroides, Actinobacteria, Verrucomicrobia, Fusobacteria; Bacteroides, Streptococcus, Faecalibacterium, and Prevotella9*
		Probiotics supplemented quadruple therapy (amoxicillin 1000 mg + clarithromycin 500 mg + esomeprazole 20 mg + potassium bismuth citrate 200 mg, twice daily for 14 days)	2 months	↓: *Proteobacteria; Helicobacter* ↑: *Firmicutes, Bacteroides, Actinobacteria, Verrucomicrobia, Fusobacteria; Bacteroides, Streptococcus, Faecalibacterium, and Prevotella9*
		Probiotics monotherapy	2 months	↓: *Firmicutes, Actinobacteria* ↑: *Proteobacteria, Bacteroides; Streptococcus Fusobacterium*
2	(Watanabe et al, 2021)	Triple therapy (a PPI [esomeprazole, lansoprazole, or rabeprazole] or vonoprazan + amoxicillin + clarithromycin, twice daily for 7 days)	13 months	↓: *Proteobacteria; Helicobacter* ↑: *Firmicutes, Bacteroides, Actinobacteria, Fusobacteria; Bacteroides, Streptococcus, Prevotella, Veillonella, Actinomyces, and Solobacterium*
3	(Mao et al, 2021)	Quadruple therapy (omeprazole 20 mg + bismuth pectin 200 mg + furazolidone 100 mg + amoxicillin 1000 mg, twice daily for 14 days)	4 weeks	↓: *Proteobacteria; Helicobacter* ↑: *Firmicutes, Bacteroides, Actinobacteria, Fusobacteria; Streptococcus, Bifidobacterium, Collinsella, Ruminococcus, Pseudomonas*
4	(Sung et al, 2020)	Triple therapy (omeprazole 20 mg + amoxicillin 1g + clarithromycin 500mg, twice daily for 7 days)	1 year	↓: *Helicobacter, Haemophilus, Actinobacillus, Neisseria*
5	(Shin et al, 2020)	Triple therapy (a standard dose of PPI + amoxicillin 1g + clarithromycin 500 mg, twice daily for 7-14 days)	57.4 months	↓: *Helicobacter* ↑: *Acinetobacter, Actinobacteria, Bacteroides*
6	(Guo et al, 2020)	Quadruple therapy (omeprazole 20 mg [twice daily for 10 days] + tetracycline 750 mg [three times daily for 10 days] + metronidazole 400 mg [three times daily for 10 days] and bismuth citrate 300 mg [twice daily for 10 days])	6 months	↓: *Proteobacteria; Helicobacter* ↑: *Cyanobacteria/Chloroplast, Bacteroidetes, Fusobacteria, Actinobacteria, Firmicutes, unknown_ Bacteria; unknown_Neisseriaceae, Staphylococcus, Corynebacterium, Fusobacterium, Bacteroides, Streptophyta, unknown_Prevotellaceae, Prevotella, Gemella, Porphyromonas, Alloprevotella, Veillonella, Neisseria, Streptococcus, Rothia, unknown_Bacteria, Haemophilus*
8	(He et al, 2019)	Quadruple therapy (esomeprazole 20 mg + bismuth subcitrate 220 mg + amoxicillin 1g + furazolidone 100 mg, twice daily for 14 days)	26 weeks	↓: *Proteobacteria; Helicobacter* ↑: *Bacteroidetes, Firmicutes; Lactobacillus, Prevotella_9, Bifidobacterium*
9	(Li et al, 2017)	Triple therapy (esomeprazole 20 mg + amoxicillin 1g + clarithromycin 500 mg, twice daily for 7 days)	8 weeks	↓: *Proteobacteria; Helicobacter* ↑: *Bacteroidetes, Fusobacteria and Actinobacteria; non- Helicobacter Proteobacteria*

↑, increased; ↓, decreased.

### Effect of *H. pylori* Eradication on Gastric Microbial Interactions

Three studies (Guo et al., 2020; Sung et al., 2020; Yuan et al., 2021) did microbial interaction analysis of gastric microbiota (**Table 4**). The study (Guo et al., 2020) evaluating quadruple therapy showed that weaker correlation strengths of gastric genera were observed 6 months after eradication. Similarly, for triple therapy, less microbial co-occurrences were found 1 year after therapy (Sung et al., 2020). Additionally, Yuan Z et al. evaluated alterations of microbial interactions following probiotics monotherapy and reported that the microbial correlation network was significantly enhanced and negative correlations between *Helicobacter* and several genera were newly emerged (Yuan et al., 2021).

**Table 4:** Major findings of gastric microbial interactions and microbiota functions.

No.	Study	Therapy	Follow-up	Microbial interactions	Microbiota functions
1	(Yuan et al, 2021)	Quadruple therapy (amoxicillin 1000 mg + clarithromycin 500 mg + esomeprazole 20 mg + potassium bismuth citrate 200 mg, twice daily for 14 days)	2 months	Not reported	Not reported
		Probiotics supplemented quadruple therapy (amoxicillin 1000 mg + clarithromycin 500 mg + esomeprazole 20 mg + potassium bismuth citrate 200 mg, twice daily for 14 days)	2 months	Not reported	Not reported
		Probiotics monotherapy	2 months	The microbial correlation network was significantly enhanced after probiotics monotherapy, when the correlation between *Helicobacter* and *Megamonas*, *Ruminococcus-1*, *Enterobacter*, *Skermanella* no longer existed, while negative correlations between *Helicobacter* and *Actinomyces*, *Rothia*, *Peptostreptococcus*, *Prevotella7*, *Veillonella*, and *Actinobacillus* were newly emerged	Not reported
4	(Sung et al, 2020)	Triple therapy (omeprazole 20 mg + amoxicillin 1g + clarithromycin 500mg, twice daily for 7 days)	1 year	Less microbial co-occurrences were found after eradication	Up-regulated after eradication: amino acid metabolism, inositol phosphate metabolism, etc. Down-regulated after eradication: folate biosynthesis, NOD-like receptor signaling, etc.
6	(Guo et al, 2020)	Quadruple therapy (omeprazole 20 mg [twice daily for 10 days] + tetracycline 750 mg [three times daily for 10 days] + metronidazole 400 mg [three times daily for 10 days] and bismuth citrate 300 mg [twice daily for 10 days])	6 months	Weaker correlation strengths of gastric genera were observed after eradication	Up-regulated after eradication: protein digestion and absorption, glycosaminoglycan degradation, gastric acid secretion, carbohydrate digestion and absorption, etc. Down-regulated after eradication: epithelial cell signaling in *H. pylori* infection, flagellar assembly, bacterial chemotaxis, bacterial secretion system, etc.
8	(He et al, 2019)	Quadruple therapy (esomeprazole 20 mg + bismuth subcitrate 220 mg + amoxicillin 1g + furazolidone 100 mg, twice daily for 14 days)	6 weeks	Not reported	Up-regulated after eradication: ABC transporters, transcription factors, butanoate metabolism, etc. Down-regulated after eradication: bacterial motility proteins, bacterial chemotaxis, flagellar assembly, etc.
			26 weeks	Not reported	Up-regulated after eradication: ABC transporters, transcription factors, DNA repair and recombination proteins, etc. Down-regulated after eradication: bacterial motility proteins, bacterial chemotaxis, flagellar assembly, etc.

### Effect of *H. pylori* Eradication on Gastric Microbiota Functions

There were three studies (He et al., 2019; Guo et al., 2020; Sung et al., 2020) that performed bioinformatic analyses to evaluate changes of gastric microbiota functions after eradication (**Table 4**). These studies yielded different findings for microbial functions, and we summarize these results. To sum up, the bacteria reproduction related pathways down-regulated, such as bacterial chemotaxis (He et al., 2019; Guo et al., 2020), flagellar assembly (He et al., 2019; Guo et al., 2020), NOD-like receptor signaling (Sung et al., 2020), etc.; and normal gastric function related pathways up-regulated, such as gastric acid secretion (Guo et al., 2020), protein digestion and absorption (Guo et al., 2020), amino acid metabolism (Sung et al., 2020), etc.

### Comparison of Gastric Microbiota Between After Eradication and Uninfected Status

Further, we summarize major findings based on seven studies (Li et al., 2017; Serrano et al., 2019; Guo et al., 2020; Shin et al., 2020; Mao et al., 2021; Watanabe et al., 2021; Yuan et al., 2021) regarding whether post-eradication gastric microbiota were restored to uninfected status (**Table 5**). For quadruple therapy, gastric microbiota that 2 months after eradication showed higher alpha diversity compared with uninfected status (Mao et al., 2021; Yuan et al., 2021); 6 months following eradication, the alpha diversity was similar to that of uninfected status (Guo et al., 2020). In terms of triple therapy, 2-month post-eradication gastric microbiota showed similar alpha diversity, beta diversity, and microbial interactions compared with uninfected status (Li et al., 2017; Serrano et al., 2019). For longer follow-up, lower alpha diversity and significant different beta diversity were reported (Watanabe et al., 2021); a recent study indicated that community structure restored to uninfected status among those without *Acinetobacter* predominance (Shin et al., 2020).

**Table 5:** Comparison of gastric microbiota between after eradication and uninfected status.

No.	Study	Therapy	Follow-up	Major findings
1	(Yuan et al, 2021)	Quadruple therapy (amoxicillin 1000 mg + clarithromycin 500 mg + esomeprazole 20 mg + potassium bismuth citrate 200 mg, twice daily for 14 days)	2 months	Higher alpha diversity and significantly different community structure compared with uninfected status
		Probiotics supplemented quadruple therapy (amoxicillin 1000 mg + clarithromycin 500 mg + esomeprazole 20 mg + potassium bismuth citrate 200 mg, twice daily for 14 days)	2 months	Higher alpha diversity and significantly different community structure compared with uninfected status
		Probiotics monotherapy	2 months	Higher alpha diversity and significantly different community structure compared with uninfected status
2	(Watanabe et al, 2021)	Triple therapy (a PPI [esomeprazole, lansoprazole, or rabeprazole] or vonoprazan + amoxicillin + clarithromycin, twice daily for 7 days)	13 months	Lower alpha diversity and significantly different community structure compared with uninfected status
3	(Mao et al, 2021)	Quadruple therapy (omeprazole 20 mg + bismuth pectin 200 mg + furazolidone 100 mg + amoxicillin 1000 mg, twice daily for 14 days)	4 weeks	Higher alpha diversity and similar community structure compared with uninfected status
5	(Shin et al, 2020)	Triple therapy (a standard dose of PPI + amoxicillin 1g + clarithromycin 500 mg, twice daily for 7-14 days)	57.4 months	The consequences of eradication can be clustered into two groups: eradicated without *Acinetobacter* predominance group (community structure restored to uninfected status) *vs.* eradicated with *Acinetobacter* predominance group (community structure did not restore to uninfected status)
6	(Guo et al, 2020)	Quadruple therapy (omeprazole 20 mg [twice daily for 10 days] + tetracycline 750 mg [three times daily for 10 days] + metronidazole 400 mg [three times daily for 10 days] and bismuth citrate 300 mg [twice daily for 10 days])	6 months	Similar alpha diversity and significantly different community structure compared with uninfected status
7	(Serrano et al, 2019)	Triple therapy (amoxicillin + clarithromycin + omeprazole, for 14 days)	2 months	Similar community structure compared with uninfected status
9	(Li et al, 2017)	Triple therapy (esomeprazole 20 mg + amoxicillin 1g + clarithromycin 500 mg, twice daily for 7 days)	8 weeks	Similar alpha diversity and similar microbial interactions compared with uninfected status

## Discussion

Our study evaluated the influence of successful *H. pylori* eradication on human gastric microbiota through systematic review and meta-analysis. Overall, no significant differences were observed between different types of therapy (quadruple therapy *vs.* triple therapy) and follow-up period (short-term *vs.* long-term). We found that alpha diversity increased in the short-term and persisted in the long-term follow-up. The microbial composition reshaped after eradication; *H. pylori*-related taxa were depleted and gastric commonly dominant commensals were enriched. For functions, bacteria reproduction related pathways down-regulated and normal gastric function related pathways up-regulated. Similar trends of alteration of gastric microbiota were observed in both quadruple therapy and triple therapy. Regarding whether eradication could restore gastric microbiota to a similar status of uninfected individuals, the results remained controversial.

*H. pylori* is a critical pathogen for GC and has been classified by the WHO-IARC as a type I carcinogen (WHO-IARC, 1994). *H. pylori* colonizes in the stomach and becomes the predominant microbe of gastric microbiota once infection occurs, leading to the dysbiosis of gastric microbiota (Engstrand and Lindberg, 2013). The direct manifestation decreases microbial diversity, which is commonly recognized as an important marker of microbial dysbiosis (Claesson et al., 2017). Further, GC and precancerous gastric lesions show decreased diversity of gastric microbiota, as well (Coker et al., 2017; Ferreira et al., 2018). Our study found that eradication of *H. pylori* could successfully increase the alpha diversity both in quadruple therapy and triple therapy; the long-term persistence of increased diversity was also seen (Li et al., 2017; He et al., 2019; Serrano et al., 2019; Guo et al., 2020; Shin et al., 2020; Sung et al., 2020; Mao et al., 2021; Watanabe et al., 2021; Yuan et al., 2021). This meta-analysis of alpha diversity is the first to report detailed changes of gastric microbiota alpha diversity after *H. pylori* eradication. In addition, significant differences of beta diversity were also observed following eradication of *H. pylori* (He et al., 2019; Guo et al., 2020; Shin et al., 2020; Sung et al., 2020; Mao et al., 2021; Watanabe et al., 2021; Yuan et al., 2021). These findings implied that eradication of *H. pylori* could reverse the impact, i.e., reduced alpha diversity, etc., of *H. pylori* infection on microbial diversity.

The human stomach was once considered to be a sterile organ owing to its strong acid production, however, the discovery of *H. pylori* and other microbes identified in stomach have changed this notion (Ozbey et al., 2020). Based on measurements using culture-independent methods, the healthy human stomach has a unique microbiota composition, with major microbes of *Firmicutes*, *Bacteroidetes*, *Actinobacteria*, *Fusobacteria*, *Proteobacteria*, etc. (Nardone and Compare, 2015). Once infected by *H. pylori*, the stomach is dominated by *H. pylori* and the abundance of other non-*H. pylori* bacterial flora decreased dramatically (Schulz et al., 2016; Schulz et al., 2018). Accordingly, the presence of *H. pylori* diminishes the commonly dominant commensals in the stomach. For the gastric microbiota composition following *H. pylori* eradication, our study shows that *H. pylori*-related taxa at different levels were depleted and gastric commonly dominant commensals were enriched (Li et al., 2017; He et al., 2019; Guo et al., 2020; Shin et al., 2020; Sung et al., 2020; Mao et al., 2021; Watanabe et al., 2021; Yuan et al., 2021), such as *Firmicutes*, *Bacteroides*, *Actinobacteria*, etc. Further, the decrease of gastric commonly dominant commensals was observed in gastric microbiota of GC and precancerous gastric lesions, indicating that the decrease of gastric commonly dominant commensals may be a risk factor for GC (Coker et al., 2017; Ferreira et al., 2018; Kadeerhan et al., 2021). It has been confirmed that the eradication of *H. pylori* could reduce GC risk (You et al., 2006; Ma et al., 2012; Ellis et al., 2019). Once *H. pylori* was eradicated, the abundance of gastric commonly dominant commensals increased, which may contribute to the reduction of GC risk. However, available studies evaluating gastric carcinogenesis related microbiota are case-control studies with relatively small sample size; cohort studies ware limited. Further large cohort studies with long-term follow-up, which evaluate the association between gastric commonly dominant commensals and GC risk, are needed.

For the interactions among microbes of gastric microbiota, *H. pylori* has negative interactions with other microbes and other microbes interacted positively with each other (Das et al., 2017; Guo et al., 2020). During the gastric lesion development, increasing strengths of gastric microbiota interactions were observed with disease progression (Coker et al., 2017; Guo et al., 2020). The INS-GAS mice study showed that INS-GAS mice with complex gastric microbiota caused more severe gastric lesions and early onset of gastrointestinal intraepithelial neoplasia (Lofgren et al., 2011). Our study demonstrates that less microbial co-occurrences were found after eradication of *H. pylori* (Guo et al., 2020; Sung et al., 2020; Yuan et al., 2021). These findings imply that stronger gastric microbiota interactions may be involved in gastric carcinogenesis.

The present study found that bacteria reproduction related pathways down-regulated and normal gastric function related pathways up-regulated in terms of bacterial function (He et al., 2019; Guo et al., 2020; Sung et al., 2020). The down-regulation of bacteria reproduction related pathways, such as bacterial chemotaxis (He et al., 2019; Guo et al., 2020), flagellar assembly (He et al., 2019; Guo et al., 2020), were consistent with the removal of *H. pylori* by eradication therapy. Notably, the infection of *H. pylori* causes suppression of gastric acid secretion (Smolka and Backert, 2012; Hammond et al., 2015) and the function of gastric acid secretion could recover following eradication (Guo et al., 2020). The gastric H^+^/K^+^-ATPase mRNA expression was markedly restored after eradication of *H. pylori*, suggesting that the restoration of H^+^/K^+^-ATPase expression may be critical in gastric acid secretion recovery (Osawa et al., 2006). These findings demonstrate that eradication of *H. pylori* contributes to the recovery of normal gastric functions.

Whether the post-eradication gastric microbiota restored to uninfected status is another issue of concern. Based on available literature enrolled in the present study, the findings remained controversial across studies. The difference between conflicting results might be explained by differences in study population, methods of sample collection, methods of sequencing, etc., between studies. Further well-designed studies are needed to address this issue.

### Strengths and Limitations

To the best of our knowledge, the present study is the first systematic review and meta-analysis to evaluate alterations of human gastric microbiota following successful *H. pylori* eradication. We reported the changes of alpha diversity indexes, beta diversity, microbial composition, microbiota functions, and microbial interactions based on the nine enrolled studies.

We acknowledge some limitations. First, the present meta-analysis has a limited sample size for some certain outcomes. Meanwhile, not all indexes were enrolled for the meta-analysis due to the heterogeneities in methods of measurement. Further, marked high heterogeneity was seen in the results of alpha diversity index. The heterogeneity across studies might be explained by differences in study population (different country, different age group, etc.), methods of sample collection, etc., between studies. Additionally, our study is a literature-based meta-analysis and the lack of access to unpublished data may lead to potential bias. What’s more, most of the enrolled studies were conducted in China and the extrapolation to other populations requires cautious interpretation.

## Conclusion

In conclusion, our study reported that successful *H. pylori* eradication could reverse the gastric microbiota dysbiosis and showed beneficial effects on gastric microbiota in terms of microbial diversity, community structure, composition, etc. Our findings may provide new insight for exploring the role of *H. pylori* and the whole gastric microbiota in gastric carcinogenesis.

## Data Availability Statement

The original contributions presented in the study are included in the article/**Supplementary Material**. Further inquiries can be directed to the corresponding author.

## AUTHOR CONTRIBUTIONS

GY and BY structured and designed the study. GY, X-SC, and G-YG performed the literature search, data extraction, did the quality assessment of included studies, and data analyses. GY wrote the first draft of the manuscript. BY and MZ critically reviewed the manuscript. All authors approved the final version.

## Funding

This work was supported by the National Natural Science Foundation of China (No. 82103727), the fellowship of China Postdoctoral Science Foundation (No. 2021M702221), Guangdong Basic and Applied Basic Research Foundation (No. 2022A1515010957), Shenzhen Sanming Project (No. SZSM201812059), Shenzhen Key Medical Discipline Construction Fund (No. SZXK040), Shenzhen Science and Technology Program (No. RCBS20210706092408008), the Scientific Research Foundation of PEKING UNIVERSITY SHENZHEN HOSPITAL (No. KYQD2021039), and Incubation Fund of Vanke School of Public Health, Tsinghua University (No. 2021PY002).

## Conflict of Interest

The authors declare that the research was conducted in the absence of any commercial or financial relationships that could be construed as a potential conflict of interest.

## Publisher’s Note

All claims expressed in this article are solely those of the authors and do not necessarily represent those of their affiliated organizations, or those of the publisher, the editors and the reviewers. Any product that may be evaluated in this article, or claim that may be made by its manufacturer, is not guaranteed or endorsed by the publisher.
