# Supplementary material for: Effect of Helicobacter Pylori Eradication on Human Gastric Microbiota: A Systematic Review and Meta-Analysis
Source: Front Cell Infect Microbiol. 2022 May 4;12:899248. doi: 10.3389/fcimb.2022.899248 (PMC9114356; doi:10.3389/fcimb.2022.899248)
Supplement: Supplementary file 1 [file Table_1.docx]

Supplementary Material

## Supplementary Table 1. Literature search strategy

| **Database** | **Search strategy** |
| --- | --- |
| PubMed | ((microbiota [Mesh]) OR "microbiome"[Title/Abstract] OR "microbiota"[Title/Abstract] OR "microﬂora"[Title/Abstract] OR "bacterial ﬂora"[Title/Abstract] OR "bacterial community"[Title/Abstract])) AND (("Helicobacter pylori"[Mesh]) OR ("Helicobacter pylori"[Title/Abstract]) OR ("H pylori "[Title/Abstract])) AND (("eradicate"[Title/Abstract]) OR ("eradication"[Title/Abstract]) OR ("treatment"[Title/Abstract]) OR ("therapy"[Title/Abstract])) |
| Web of Science | (TS=(microbiome) OR TS=(microbiota) OR TS=(microﬂora) OR TS=(“bacterial ﬂora”) OR TS=(“bacterial community”) ) AND (TS=(“Helicobacter pylori”) OR TS=(“H pylori”)) AND (TS=(“eradicate”) OR TS=(“eradication”) OR TS=(“treatment”) OR TS=(“therapy”)) |
| EMBASE | ('microbiome':ti,ab,kw OR 'microbiota':ti,ab,kw OR 'microﬂora':ti,ab,kw OR 'bacterial ﬂora':ti,ab,kw OR 'bacterial community':ti,ab,kw) AND ('Helicobacter pylori'/exp OR 'Helicobacter pylori':ti,ab,kw OR 'H pylori':ti,ab,kw )AND ('eradicate':ti,ab,kw OR 'eradication':ti,ab,kw OR 'treatment':ti,ab,kw OR 'therapy':ti,ab,kw) |

Systematic search was performed on November 27, 2021

## Supplementary Table 2. Data extraction form

**I. Basic information of included studies**

| Authors: _______________________________ |
| --- |
| Publication year: _________________________ |
| Journal: ________________________________ |
| Title: __________________________________ |
| Region: ________________________________ |
| Aims: _________________________________ |
| Sample size: _____________________________ |

**II. Interventions and comparisons**

| *H. pylori* eradication therapy:  □bismuth-based quadruple therapy  □triple therapy  □other: ______________ |
| --- |
| Follow-up time:  □1st follow-up: ______________  □2nd follow-up: ______________  □3rd follow-up: ______________ |

**III. Outcomes**

| Evaluation method of microbiota:  □16S rRNA gene sequencing  □Metagenomic sequencing  □Viral sequencing  □Cultivation  □Other _______ |
| --- |
| Indexes used for alpha diversity:  □Shannon □Chao 1 □Observed species  □Simpson □Pielou evenness □Sobs index  □ACE □Phylogenetic diversity □Other _______ |
| Major findings about alpha diversity: ______________ |
| Major findings about beta diversity: ______________ |
| Major findings about differential microbes:  □Phylum: ______________  □Genus: ______________ |
| Major findings about microbiota functions: ______________ |
| Major findings about microbial interactions: ______________ |

**IV. Information of data extraction**

| Reviewer name: ______________ |
| --- |
| Date of data extraction: ______________ |

## Supplementary Table 3. Methodological index for non-randomized studies (MINORS)

| **Major Components** | **Response options** | | |
| --- | --- | --- | --- |
| 1. A clearly stated aim | Not reported (0 point) | Reported but inadequate (1 point) | Reported and adequate (2 point) |
| 2. Inclusion of consecutive patients | Not reported (0 point) | Reported but inadequate (1 point) | Reported and adequate (2 point) |
| 3. Prospective collection of data | Not reported (0 point) | Reported but inadequate (1 point) | Reported and adequate (2 point) |
| 4. Endpoints appropriate to the aim of the study | Not reported (0 point) | Reported but inadequate (1 point) | Reported and adequate (2 point) |
| 5. Unbiased assessment of the study endpoint | Not reported (0 point) | Reported but inadequate (1 point) | Reported and adequate (2 point) |
| 6. Follow-up period appropriate to the aim of the study | Not reported (0 point) | Reported but inadequate (1 point) | Reported and adequate (2 point) |
| 7. Loss to follow up less than 5% | Not reported (0 point) | Reported but inadequate (1 point) | Reported and adequate (2 point) |
| 8. Prospective calculation of the study size | Not reported (0 point) | Reported but inadequate (1 point) | Reported and adequate (2 point) |
| 9. An adequate control group | Not reported (0 point) | Reported but inadequate (1 point) | Reported and adequate (2 point) |
| 10. Contemporary groups | Not reported (0 point) | Reported but inadequate (1 point) | Reported and adequate (2 point) |
| 11. Baseline equivalence of groups | Not reported (0 point) | Reported but inadequate (1 point) | Reported and adequate (2 point) |
| 12. Adequate statistical analyses | Not reported (0 point) | Reported but inadequate (1 point) | Reported and adequate (2 point) |
| **Total score** |  | | |

## Supplementary Table 4. Cochrane risk-of-bias tool for randomized trials (RoB 2)

| **Bias domain and signaling question** | **Response options** | | |
| --- | --- | --- | --- |
|  | **Lower risk of bias** | **Higher risk of bias** | **Other** |
| **1. Bias arising from the randomization process** | | | |
| 1.1 Was the allocation sequence random? | Yes/ Probably Yes | No/ Probably No | No Information |
| 1.2 Was the allocation sequence concealed until participants were enrolled and assigned to interventions? | Yes/ Probably Yes | No/ Probably No | No Information |
| 1.3 Did baseline differences between intervention groups suggest a problem with the randomization process? | No/ Probably No | Yes/ Probably Yes | No Information |
| *Risk-of-bias judgment (low/high/some concerns)* |  | | |
| *Optional: What is the predicted direction of bias arising from the randomization process?* |  | | |
| **2. Bias due to deviations from intended interventions** | | | |
| 2.1 Were participants aware of their assigned intervention during the trial? | No/ Probably No | Yes/ Probably Yes | No Information |
| 2.2 Were carers and people delivering the interventions aware of participants’ assigned intervention during the trial? | No/ Probably No | Yes/ Probably Yes | No Information |
| 2.3 If Yes/ Probably Yes/ No Information to 2.1 or 2.2: Were there deviations from the intended intervention that arose because of the trial context? | No/ Probably No | Yes/ Probably Yes | No Information/ Not Applicable |
| 2.4 If Yes/ Probably Yes/ No Information to 2.3: Were these deviations likely to have affected the outcome? | No/ Probably No | Yes/ Probably Yes | No Information/ Not Applicable |
| 2.5 If Yes/ Probably Yes to 2.4: Were these deviations from intended intervention balanced between groups? | Yes/ Probably Yes | No/ Probably No | No Information/ Not Applicable |
| 2.6 Was an appropriate analysis used to estimate the effect of assignment to intervention? | Yes/ Probably Yes | No/ Probably No | No Information |
| 2.7 If No/ Probably No/ No Information to 2.6: Was there potential for a substantial impact (on the result) of the failure to analyses participants in the group to which they were randomized? | No/ Probably No | Yes/ Probably Yes | No Information/ Not Applicable |
| *Risk-of-bias judgment (low/high/some concerns)* |  | | |
| *Optional: What is the predicted direction of bias due to deviations from intended interventions?* |  | | |
| **3. Bias due to missing outcome data** | | | |
| 3.1 Were data for this outcome available for all, or nearly all, participants randomized? | Yes/ Probably Yes | No/ Probably No | No Information |
| 3.2 If No/ Probably No/ No Information to 3.1: Is there evidence that the result was not biased by missing outcome data? | Yes/ Probably Yes | No/ Probably No | Not Applicable |
| 3.3 If No/ Probably No to 3.2: Could missingness in the outcome depend on its true value? | No/ Probably No | Yes/ Probably Yes | No Information/ Not Applicable |
| 3.4 If Yes/ Probably Yes/ No Information to 3.3: Is it likely that missingness in the outcome depended on its true value? | No/ Probably No | Yes/ Probably Yes | No Information/ Not Applicable |
| *Risk-of-bias judgment (low/high/some concerns)* |  | | |
| *Optional: What is the predicted direction of bias due to missing outcome data?* |  | | |
| **4. Bias in measurement of the outcome** | | | |
| 4.1 Was the method of measuring the outcome inappropriate? | No/ Probably No | Yes/ Probably Yes | No Information |
| 4.2 Could measurement or ascertainment of the outcome have differed between intervention groups? | No/ Probably No | Yes/ Probably Yes | No Information |
| 4.3 If No/ Probably No/ No Information to 4.1 and 4.2: Were outcome assessors aware of the intervention received by study participants? | No/ Probably No | Yes/ Probably Yes | No Information |
| 4.4 If Yes/ Probably Yes/ No Information to 4.3: Could assessment of the outcome have been influenced by knowledge of intervention received? | No/ Probably No | Yes/ Probably Yes | No Information/ Not Applicable |
| 4.5 If Yes/ Probably Yes/ No Information to 4.4: Is it likely that assessment of the outcome was influenced by knowledge of intervention received? | No/ Probably No | Yes/ Probably Yes | No Information |
| *Risk-of-bias judgment (low/high/some concerns)* |  | | |
| *Optional: What is the predicted direction of bias in measurement of the outcome?* |  | | |
| **5. Bias in selection of the reported result** | | | |
| 5.1 Were the data that produced this result analyzed in accordance with a prespecified analysis plan that was finalized before unblinded outcome data were available for analysis? | Yes/ Probably Yes | No/ Probably No | No Information |
| Is the numerical result being assessed likely to have been selected, on the basis of the results, from: |  |  |  |
| 5.2 ... multiple eligible outcome measurements (eg, scales, definitions, time points) within the outcome domain? | No/ Probably No | Yes/ Probably Yes | No Information |
| 5.3 ... multiple eligible analyses of the data? | No/ Probably No | Yes/ Probably Yes | No Information |
| *Risk-of-bias judgment (low/high/some concerns)* |  | | |
| *Optional: What is the predicted direction bias due to selection of the reported results?* |  | | |
| **6. Overall bias** | | | |
| *Risk-of-bias judgment (low/high/some concerns)* |  | | |
| *Optional: What is the overall predicted direction of bias for this outcome?* |  | | |
